# Production losses five months after outbreak with a recombinant of two PRRSV vaccine strains in 13 Danish sow herds

**DOI:** 10.1186/s40813-020-00165-z

**Published:** 2020-10-06

**Authors:** C. S. Kristensen, M. G. Christiansen, K. Pedersen, L. E. Larsen

**Affiliations:** 1 SEGES Danish Pig Research Centre, Copenhagen, Denmark; 2 Department of Veterinary and Animal Sciences, University of Copenhagen, Frederiksberg, Denmark

**Keywords:** PRRSV, Production loss, Stillborn, Pre-weaning mortality

## Abstract

**Background:**

In July 2019, a PRRSV-negative boar station was infected with a recombinant of two PRRSV vaccine strains, which subsequently spread to at least 36 herds that had received semen from the boar station. In the following months, all the infected herds reported reduced productivity. The aim of the present study was to evaluate the impact of the PRRS outbreak.

**Results:**

Production data were collected from 13 of the herds. The average levels of farrowings/week, liveborns/litter, stillborns/litter, pre-weaning mortality and weaned pigs/litter were compared for the five-month period after infection and the preceding 7 months before infection with the new variant of PRRSV-1. Twelve herds experienced a decrease in farrowings/week (0.1–10.8% fewer farrowings/week), and all herds experienced fewer liveborns (0.8–4.8 fewer liveborns/litter) and more stillborns (0.6–2.6 more stillborns/litter). Pre-weaning mortality nearly doubled in half of the herds. Overall, the 13 herds were missing 2.4–6.5 pigs/litter at weaning during the 5 months after infection compared to the seven preceding months before infection.

**Conclusion:**

In this study, the impact of this new PRRSV-1 variant on productivity exceeded that typically seen in Danish herds infected with PRRSV-1.

## Background

Since it first appeared at the beginning of the 1990s, porcine reproductive and respiratory syndrome virus (PRRSV) has been one of the major health challenges in pig production. According to figures from the Danish SPF society, approximately 35% of Danish pig herds are positive for antibodies to PRRSV, although the prevalence of serologically positive herds is declining [1]. Both PRRSV-1 and PRRSV-2 are prevalent in Danish herds, and some herds are infected with both species.

In 1994, production losses due to acute infections with PRRS in 30 Danish herds were estimated to be 1.2 pigs/year [2]. A Danish study from 2013 found an average decrease in liveborns of 0.7 liveborns/litter, an average increase in the number of stillborns of 0.3 stillborns/litter and an average of 1.0 weaned pig less per litter [3].

Limited surveillance of the genetic diversity is practised in Denmark, but the available data indicate that, until the summer of 2019, two major clades of PRRSV-1 were co-circulating [4]. One of the clades shares a high level of genetic similarity to the Porcilis vaccine strain “DV” and probably represents a group of field viruses originating from this vaccine strain. The strains clustering in the other clade are up to 12% different from the “Porcilis-like” viruses and include the first PRRSV-1 strain isolated in Denmark in 1992 [4]. All these viruses belong to the PRRSV-1, subtype 1, whereas PRRSV-1 strains belonging to subtypes 2, 3 and 4 have never been detected in Denmark [5–7].

In July 2019, PRRSV-1 was detected in samples taken as part of the routine PRRSV surveillance in one of the Danish PRRSV-negative boar stations. More than 70 breeding and multiplier herds and up to 700 production herds were at risk of having received semen from the most likely time of infection of the boar station and until the boar station was closed. Indeed, the virus was shortly after detected in three breeding herds and in at least 33 production herds that had received semen from this station. Subjective preliminary reports from the veterinarians consulting the herds and the herd owners indicated that the virus induced clinical signs similar to, or even exceeding, those normally observed in Danish herds infected with PRRSV-1. The clinical signs included sustained reproductive failures and high piglet mortality.

The aim of the present study was to evaluate the impact of the outbreak in the infected herds over a period of 5 months after infection compared to the preceding 7 months. Furthermore, the production losses were compared to previous studies on the impact of PRRSV in Danish herds.

## Results

### Description of herds

Only 18 herd owners responded to the invitation letter, three of whom did not want to share data and two had given up recording data on productivity due to the psychological pressure caused by all the dead piglets. Therefore, we managed to include data from a total of 13 herds infected with the new PRRSV-1 variant in July 2019.

The herd size ranged from 324 to 1650 sows (1009 median). The declared health status reported by SPF-SuS [1] was negative for PRRS prior to infection for ten of the herds, one herd was positive for PRRSV-1, one herd was positive for both PRRSV-1 and PRRSV-2, and one herd had performed a partial elimination of PRRSV-2 and was in the following 6-month period before the PRRS-negative status could be obtained (Table 1). In the Danish SPF-SuS system, the negative PRRS-status is based on clinical evaluation by the veterinarian every month and 20 blood samples investigated for antibodies for PRRSV once a year. If antibodies are percent, the herd change to PRRS positive. To return from a PRRS-positive to PRRS-negative status, the herd must perform an elimination. If a partial elimination is performed, the herd will have a 6-month period, before the PRRS-negative status is obtained.

**Table 1:** The PRRSV status before infection with the new PRRSV-1 variant, and the PRRS vaccines used after infection for the 13 herds included in the study

Herd	PRRS status before infection	Use of PRRS mass vaccination after infection
A	Negative	Sows with Unistrain PRRS
B	Negative	Sows with Unistrain PRRS
C	Negative	Sows with Unistrain PRRS
D	Negative	Sows with Unistrain PRRS
E	Negative	Sows with Unistrain PRRS
F	Negative	Sows with Unistrain PRRS
G	Negative	Sows with Unistrain PRRS
H	Negative	Sows with Unistrain PRRS
I	Positive PRRSV1 + PRRSV2	Sows with Unistrain PRRS
J	Negative	Sows with Porcilis® PRRS VET
K	Positive PRRS1	Sows with Porcilis® PRRS VET
L	Under elimination of PRRSV2	Sows with Porcilis® PRRS VET
M	Negative	Sows with Progressis® Vet

Records from VETSTAT (the Danish system for surveillance of the veterinary use of drugs) showed that, prior to the infection with the recombinant PRRSV-strain, only one herd had mass-vaccinated the sows with “Porcilis® PRRS VET” (MSD Animal Health, USA). After infection with the new PRRSV-1 variant, most of the herd owners chose to mass-vaccinate with a modified live PRRS vaccine (MLV) in an attempt to control the new PRRSV-1 variant. Most of the herds used the MLV “Unistrain PRRS” (Hipra, Spain), three herds used the MLV “Porcilis® PRRS VET”, and one herd used the killed-virus vaccine “Progressis® Vet” (CEVA, France) (Table 1). The number of times the herds used mass vaccination was not evaluated.

### Productivity and impact on health

**Fig. 1:**
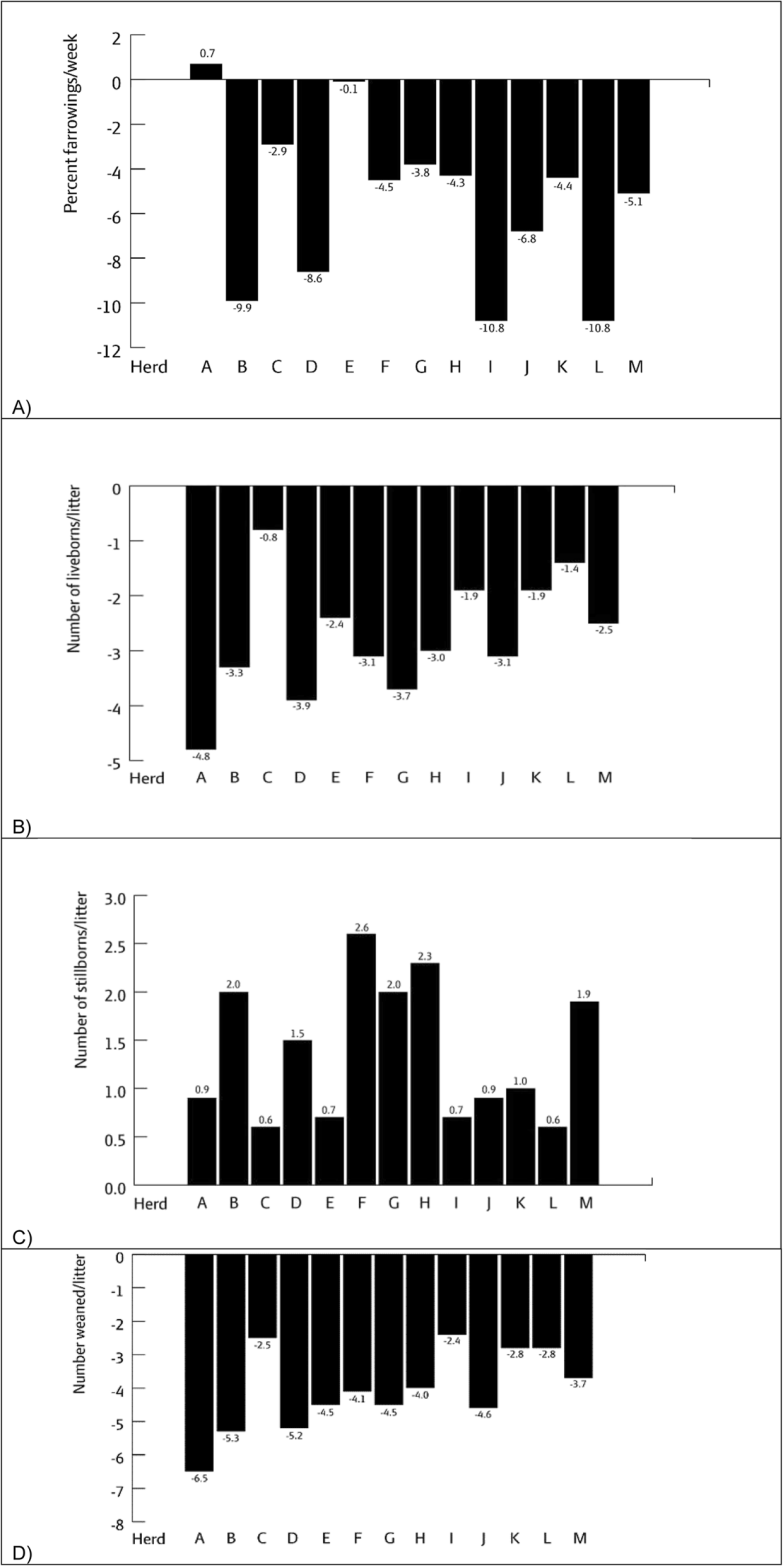
Percent change in farrowings/week (**a**) and marginal change in the number of liveborns/litter (**b**), stillborns/litter (**c**) and number weaned pigs/litter (**d**) when the five-month period after infection with the new PRRS1-variant was compared to the preceding 7 months in the 13 herds included in the study

The number of farrowings per week was affected by infection with the new PRRSV-1 variant. Two herds had 10.8% fewer farrowings per week, while one herd had 0.7% more farrowings per week when the five-month period after infection was compared to the preceding 7 months for the 13 herds (Fig. 1a). There were fewer liveborn pigs at farrowing in all 13 herds. The results revealed a decrease in the number of liveborns of 0.8–4.8 liveborns/litter after infection with the new PRRSV-1 variant in the 13 herds (Fig. 1b). The average decrease in liveborns per litter was 2.8 pigs. In addition to there being fewer liveborn pigs, there was an increase in stillborns of 0.6–2.6 stillborns/litter (Fig. 1c), corresponding to an average increase of 1.4 stillborns/litter after infection with the new PRRSV-1 variant.

The pre-weaning mortality, including stillborn piglets, increased in all 13 herds after infection with the new PRRS1-variant. Prior to the outbreak, the herds had an average pre-weaning mortality of 23% with a variation of 20 to 27%. After infection with the new PRRSV-1 variant, the pre-weaning mortality increased to an average of 40%, with a variation of 24 to 46%. Almost half of the herds experienced close to or more than a doubling of total piglet mortality after the outbreak (Table 2).

**Table 2:** Pre-weaning mortality including stillborn piglets when the five-month period after infection with the new PRRS1-variant was compared to the preceding 7 months in the 13 herds included in the study

Herd	Pre-weaning mortality before infection (%)	Pre-weaning mortality after infection (%)	Factor increase in pre-weaning mortality when comparing period before and after infection
A	24%	46%	1.92
B	20%	45%	2.27
C	22%	24%	1.09
D	23%	42%	1.85
E	31%	38%	1.22
F	24%	43%	1.80
G	26%	44%	1.74
H	23%	41%	1.78
I	27%	32%	1.15
J	22%	39%	1.73
K	20%	31%	1.57
L	22%	34%	1.56
M	27%	44%	1.66

The increased pre-weaning mortality was also reflected in the lower number of weaned pigs/litter in all 13 herds (Fig. 1d). The worst affected herd weaned 6.5 fewer pigs/litter, and the least affected herd weaned 2.4 fewer pigs/litter when the five-month period after infection was compared to the preceding 7 months.

## Discussion

The production losses in 13 Danish herds infected with the new PRRSV-1 variant have been documented in the present report. Only data from 13 of the more than 33 infected herds were obtained. Although the reason for participating or refusing to participate is not known for all the contacted herds, there is no indication that the participating herds were not representative of all the infected herds; however, this cannot be documented.

The herds included in the study had received semen from the infected boar station in July 2019 and experienced outbreaks in the following weeks. Before the outbreak, the monitoring of PRRSV at the boar stations was based on antibodies and performed every second week. After the outbreak, the surveillance was changed and now the boar stations are monitored every week based on detection of PRRSV by PCR and antibodies every second week.

The PRRSV isolated from the boar station had never been detected in Denmark or elsewhere prior to July 2019 and was therefore considered to be a new PRRS1-variant. The new PRRS1-variant is a recombination between the vaccine strains included in the Unistrain PRRS vaccine (Hipra, Spain) and the Suvaxzyn PRRSV (Zoetis Animal Health, USA) in that the ORF 1, ORF 2 and part of ORF 3 are more than 99% identical to the Suvaxyn strain, and the rest of the genome is more than 99% identical to the Unistrain vaccine strain. The breakpoint is located in ORF 3 encoding the glycoprotein 3 (GP3) after nucleotide position 201, corresponding to amino acid 67 [8]. It is not the first time that a recombinant of two modified live PRRSV vaccines has been documented [9]. Only herds in which the virus shared > 99% homology with the Zoetis vaccine strain in ORF 2 and > 99% identity with the Unistrain strain in ORF 5 were regarded as being infected through semen with the new variant strain and were included in the study.

For ten of the herds, the health status reported by SPF-SuS [1] was negative for PRRS prior to infection. Due to the low number of herds included in the study, the production losses cannot be compared for herds that were positive or negative to PRRS before infection with the new variant. Based on the visual inspection of the data, there do not appear to be any differences in the severity between herds that were positive or negative prior to the outbreaks.

Records from VETSTAT [10] show that, prior to PRRS infection, only one of the PRRS-positive herds (K) used mass vaccination against PRRSV prior to the outbreak. Despite this vaccination of all sows, the herd recorded production loses after infection with the recombinant PRRSV. After the outbreaks, most of the herd owners chose to vaccinate all sows, some several times, with a modified live PRRS vaccine. Nine of the 13 herds used “Unistrain PRRS”, since the new PRRSV-1 variant is almost identical to the Unistrain vaccine strain in ORF 5, which is considered to be the major target for neutralising antibodies [11]. Again, due to the low number of herds included in the study, the production losses cannot be compared with regard to the selected PRRS vaccine, although, based on the visual inspection of the data, there do not appear to be any differences. Since all herds decided to use mass vaccination against PRRSV, it cannot be stated whether this use of vaccines decreased or increased the production loses.

The number of farrowings per week was affected by infection with the new PRRSV-1 variant, with 12 herds experiencing a reduction, which is expected after infection with PRRSV. One herd had a small increase in farrowings per week, which was probably due to the fact that relatively more sows were inseminated after infection in order to compensate for a potential decrease in farrowings as a result of PRRSV.

Since PRRSV affects both the survival of the piglets and the reproduction of the sow, it is still too early to analyse the overall effects of PRRSV-1 infection on the reproductive parameters (e.g. wastage days and farrowing percentage), and therefore only effects including farrowings/week, liveborns/week, stillborns/week, pre-weaning mortality and weaned pigs/litter were included in the analysis.

The closest study to the one reported here seems to be the Danish study from 2013, which included eight herds. Seven herds were previously PRRS-free, and, of these, three were infected with PRRSV-1 and four with PRRSV-2. One herd had previously been infected with PRRSV-1 and had experienced an acute outbreak after introduction of PRRSV-2. The period with reduced production was estimated to be between ten and 90 weeks [3].

All 13 herds experienced fewer liveborn pigs at farrowing, which was also expected after an outbreak of PRRS. The average decrease in liveborn pigs per litter was 2.8 pigs for every litter during the five-month period after infection compared to the preceding 7 months. When comparing this with the previous Danish estimate from 2013 [3], where the decrease was 0.7 liveborns/litter based on a seven-month period after infection with PRRSV, it is seen that this new PRRSV-1 variant results in higher production losses than older variants of PRRSV seen in Denmark. Even if it was assumed that the 13 herds went back to normal productivity for the last 2 months, so that the period after infection was 7 months (as in the study from 2013), this new PRRS1-variant would still result in a decrease in liveborns of more than two liveborn pigs per litter.

In addition to there being fewer liveborn pigs, there was also an average increase in stillborn pigs per litter of 1.4 for the 5 months after infection compared to the preceding 7 months. In the 2013 study, there was an average increase in stillborns per litter of just 0.3 during the seven-month period after infection with PRRSV. Thus, infection with the new PRRS1-variant appears to result in one more stillborn pig per litter compared to the previous outbreaks with other variants.

The pre-weaning mortality increased to an average of 40% after infection with the new PRRSV-1 variant. The increased pre-weaning mortality was also reflected in a lower number of weaned pigs from each litter in all 13 herds. On average, the 13 herds were missing 4.1 pigs from each litter at weaning when the five-month period after infection was compared to the preceding 7 months. In the 2013 study, the eight herds were missing an average of only 1.0 weaned pig from each litter. Again, this new variant seems to have increased the negative impact on production parameters compared to other PRRSV variants previously seen in Denmark.

A reduction in liveborns per litter of between one and two pigs has been reported in several studies after infection with PRRSV [12–17]. Similarly, an increased pre-weaning mortality of 4 to 17% [12–14, 16–18] and a decreased farrowing rate [19–21] have been reported from outbreaks in other countries. As a result of this decline in farrowings and increased piglet mortality, the number of weaned pigs per litter decreased by two to three pigs [12, 15, 20]. Differences in the virulence of circulating viruses, the presence of both PRRSV-1 and PRRSV-2 and differences in production systems and general health status make it difficult to compare studies of production losses resulting from the introduction of PRRSV in different regions/countries. Comparisons are even more difficult to make, since different countries have different ways of calculating and reporting data. Despite these limitations, it is clear that the losses seen in connection with this new recombinant PRRS1-variant exceeded not only the losses normally seen in Danish PRRSV-infected herds but also the losses seen in connection with PRRS outbreaks in other regions and countries.

## Conclusions

Infection with a new recombinant PRRSV-1 variant in 13 herds resulted in a huge decrease in productivity during the subsequent five-month period. Based on the assessment of the data, it can be concluded that the impact of this new PRRSV-1 variant exceeds that previously encountered in Denmark and also exceeds that reported in other countries.

## Methods

### Herds included

Herd owners of herds diagnosed with the new PRRSV-1 variant were contacted via letter and invited to share their production data from 2019. Breeding herds were omitted, since they all completed an elimination programme shortly after infection with the new PRRS1-variant. Only herds that had obtained semen from the infected boar station and sequenced the recombinant of the two PRRSV vaccine strains were included in the study. The identity of the virus was verified by sequencing of samples from individual animals. The sequenced part included at least the full ORF 5 gene and the full ORF 2 gene, but in most cases, the full ORFs 2–7 were sequenced. Only herds in which the virus shared > 99% homology with the Zoetic vaccine strain in ORF 2 and > 99% identity with the Unistrain strain in ORF 5 were regarded as being infected with the recombinant strain and were included in the study. Details on the laboratory methods used have previously been described [8].

To anonymise the inventory, details on the herd size and health status have been omitted from this report.

Productivity data from 2018 and 2019 were provided by the herd owners. Since all herds were infected with PRRSV during the month of July, data were divided into the period preceding PRRSV-1 infection and the period after PRRSV-1 infection as follows: preceding PRRSV-1 infection: 01.12.2018–30.06.2019 and after PRRSV-1 infection: 01.08.2019–31.12.2019.

Thus, the period “preceding PRRSV-1 infection” covers 7 months, and the period “after PRRSV-1 infection” covers 5 months. The month of July was omitted, since the exact infection time in July could not be confirmed.

### Production data

Farowings/week were calculated as a percentage difference for the preceding 7 months compared to the 5 months after infection. Liveborns/litter, stillborns/litter and weaned pigs/litter were calculated nominally and descriptively as the marginal difference between the preceding 7 months and the 5 months after infection for each of the participating herds. Pre-weaning mortality, including both stillborns and liveborns that died before weaning, was calculated as a percentage for the preceding 7 months compared to the 5 months after infection.

Data on the use of PRRS vaccines in 2019 were extracted from VETSTAT [10]. Taking into account the herd size, these data were used to determine whether the herd owner should choose to mass-vaccinate the herd with a PRRS vaccine and which vaccine should be used.

The herd health status before infection with a new PRRSV-1 variant was extracted from the SPF-SuS [1] to determine whether the herd was positive or negative for PRRS prior to infection with the new PRRSV-1 variant.

## Data Availability

Besides the presented data, raw data can be shared upon reasonable request by contacting the corresponding author and will require prior acceptance from the relevant herd owners.
